# Statistical Mechanics of On-Line Learning Under Concept Drift

**DOI:** 10.3390/e20100775

**Published:** 2018-10-10

**Authors:** Michiel Straat, Fthi Abadi, Christina Göpfert, Barbara Hammer, Michael Biehl

**Affiliations:** 1 Bernoulli Institute for Mathematics, Computer Science and Artificial Intelligence, University of Groningen, Nijenborgh 9, 9747 AG Groningen, The Netherlands; 2 Center of Excellence—Cognitive Interaction Technology (CITEC), Bielefeld University, Inspiration 1, 33619 Bielefeld, Germany

**Keywords:** concept drift, on-line learning, continual learning, neural networks, learning vector quantization, statistical physics of learning

## Abstract

We introduce a modeling framework for the investigation of on-line machine learning processes in non-stationary environments. We exemplify the approach in terms of two specific model situations: In the first, we consider the learning of a classification scheme from clustered data by means of prototype-based Learning Vector Quantization (LVQ). In the second, we study the training of layered neural networks with sigmoidal activations for the purpose of regression. In both cases, the target, i.e., the classification or regression scheme, is considered to change continuously while the system is trained from a stream of labeled data. We extend and apply methods borrowed from statistical physics which have been used frequently for the exact description of training dynamics in stationary environments. Extensions of the approach allow for the computation of typical learning curves in the presence of concept drift in a variety of model situations. First results are presented and discussed for stochastic drift processes in classification and regression problems. They indicate that LVQ is capable of tracking a classification scheme under drift to a non-trivial extent. Furthermore, we show that concept drift can cause the persistence of sub-optimal plateau states in gradient based training of layered neural networks for regression.

## 1. Introduction

The many challenges of modern data science call for the design of efficient methods for automated analysis. Machine learning techniques play a key role in this context [1,2,3].

The development of modeling frameworks in which to obtain general insights into practically relevant phenomena is instrumental to achieve the necessary theoretical understanding. Analytical and computational approaches that come from or are related to statistical physics [4,5,6,7,8,9] have played an important role in this field and continue to do so.

### 1.1. Concept Drift and Continual Learning

In this contribution, we address a topic which is currently attracting increasing interest in the scientific community: the efficient training of machine learning systems in a non-stationary environment, where the target task or the statistical properties of the example data vary with time (see, for instance, [10,11,12,13,14,15] and references therein). Terms such as continual learning and lifelong learning have been coined in this context.

Frequently, the set-up of machine learning processes comprises two different stages (see, for instance, [1,2,3]): In the training phase, a given set of example data is analyzed, information is extracted and a corresponding hypothesis is parameterized in terms of, e.g., a classifier or regression system. In the subsequent working phase, this hypothesis is applied to novel data. Implicitly, one assumes that the training set is representative of the problem and that statistical properties of the data and the actual target task do not change after training.

For many practical applications of machine learning, the assumption of stationarity may be well justified. However, the conceptual and temporal separation of training and working phase is not very plausible in human and other biological learning processes [16,17]. As an example, in a predator and prey system, strategies can change continuously with species trying to adapt to their adversaries’ behavior. In addition, in many technical applications of machine learning, the separation becomes inappropriate if the actual task of learning, e.g., the target classification, changes in time [10]. Moreover, very frequently, the training samples become available in the form of a stream of data (e.g., [11,12,13,14]). In such situations, the learning system must be able to detect and track concept drift, i.e., forget irrelevant, older information while continuously adapting to more recent inputs. Examples for this situation can be found, for instance, in robotics. Other problems, such as the filtering of spam messages in e-mail communication, resemble the predator–prey example in that the learning systems try to adapt to changing strategies of their opponents. Further applications range from fraud detection, quality control and customer segments management to drop out prediction for e-learning and gaming [10]. Overviews of earlier work and recent developments in the context of machine learning in non-stationary environments are provided, for instance, in [10,11,12,13,14,15]. While drift can occur in any learning scenario, in this contribution, we focus on supervised learning.

In the literature, two major types of non-stationary environments have been discussed [10,11,12,13,14,15]: In so-called virtual drifts, the statistical properties of the available example data change with time, while the actual target task, e.g., the classification or regression scheme, remains unaltered. The term real drift has been coined for situations in which the target itself is time-dependent. Frequently, real drift processes are accompanied by additional virtual drifts.

### 1.2. Models of On-Line Learning Under Concept Drift

There exists a large variety of technologies which address learning in the context of drift (see [10,11,12,13,14] for overviews). On a global level, one often differentiates so-called active methods, which aim for an explicit detection of drift and according action of the learning system, and passive methods, which can implicitly react to drift by their design. Popular active methods combine statistical tests for novelty detection [18] with a rearrangement or retraining of the system to account for the observed drift. The latter is particularly efficient if, for instance, ensemble methods are used [19,20]. The need for explicit drift detection often has the consequence that only specific types of drift can be dealt with (one exception being found in [20]). In particular, small gradual drifts are notoriously difficult to detect [21]. Passive methods continuously adapt the model according to the given data. Thus, they automatically react to all types of drift which is present in the training data. However, they face the classical stability-plasticity dilemma: relevant novel information has to be dealt with while preserving already learned signals. Local or hybrid schemes have been particularly successful in the past years (see, e.g., [21,22]). Other popular passive technologies rely on online learning schemes, in particular online gradient descent, which has been incorporated into drift learning strategies for the simple perceptron, neural networks, or extreme learning machines, as an example [23,24]. The behavior of such models varies extensively across different learning scenarios [11].

In this contribution, we study two basic scenarios of on-line learning in non-stationary environments, addressing binary classification and continuous regression problems. We present a mathematical model of drifting concepts in on-line training from high-dimensional data. Methods borrowed from statistical physics facilitate the study of the typical learning dynamics for different training scenarios and strategies. While the approach is suitable for virtual and real drift processes, here, we focus on the study of explicitly time-dependent target concepts.

With respect to classification, we consider Learning Vector Quantization (LVQ) as an example framework, i.e., prototype-based systems as originally suggested by Kohonen [25,26,27,28,29]. LVQ training is most frequently done in an on-line setting by presenting a sequence of single examples which are used to improve the system iteratively [28,29]. Therefore, LVQ should constitute a promising framework for incremental learning in the presence of concept drift.

Layered neural networks with sigmoidal, continuous activation functions serve as an example system in the context of regression. Specifically, we consider the so-called Soft Committee Machine (SCM), a shallow architecture which can be trained by means of on-line (stochastic) gradient descent [30,31,32,33,34,35,36]. Gradient based techniques are widely used also for multi-layered deep architectures and their suitability for the learning of non-stationary targets is a question of significant relevance [3,37].

### 1.3. Relation to Earlier Work

Note that several studies exist which compare different learning algorithms for streaming data experimentally (see, e.g., [11,12] and references therein). Unlike these empirical investigations, our contribution aims for a formal, mathematical framework which can abstract from the variations which occur in the course of a concrete, real world training cycle.

Methods borrowed from statistical physics have been used to analyze the typical behavior of various learning systems in model scenarios [4,5,6,7]. The particularly successful analysis of on-line learning is based on the assumption that a sequence of independently generated random *N*-dimensional examples is presented to the learning system [8,9,38]. Further simplifying assumptions and the consideration of the so-called thermodynamic limit N→∞ facilitate the exact mathematical description of typical learning curves in terms of ordinary differential equations (ODE). For detailed discussions of the limitations of the approach as well as extensions that allow to overcome them (see several contributions in [38] and, for instance, [39]).

Various reviews, article collections and monographs present and discuss the approach with respect to supervised learning in simple perceptrons and multilayered neural networks (see e.g., [4,5,6,7,8,9,38] and references therein). Similarly, the dynamics of unsupervised learning has been studied, including prototype-based competitive learning, Principal Component Analysis and related schemes [40,41,42].

Stationary model densities of clustered data, similar to the ones considered here for LVQ, have been studied with respect to several unsupervised and supervised training schemes (see [40,41,42,43,44,45] for examples and further references). Supervised LVQ training was considered more recently in the framework of simplifying model situations in [39,46,47,48,49].

The SCM in stationary environments has been studied extensively from the statistical physics perspective. Practically relevant phenomena, such as the occurrence of quasi-stationary plateau states have been investigated in great detail (see [30,31,32,33,34,35,36,38] for examples and further references).

The presence of concept drift has also been addressed within the statistical physics of on-line learning. State-of-the-art investigations have considered, in particular, the learning of time-dependent, linearly separable rules as a model system in [50,51,52,53]. Note that the assumption of statistically independent examples in the stream of data does not hinder the study of meaningful drift scenarios. It is, for instance, well possible to consider settings in which the characteristics of the generating density or the target itself depends, implicitly, on the previous training. As an example, adversarial drifts have been considered in [50,51,52,53] for the simple perceptron.

To the best of our knowledge, we present here the first statistical mechanics analysis of on-line learning under concept drift in prototype-based classification and layered neural networks for regression.

### 1.4. Outline

The main aim of this work is to present and establish a theoretical framework in which to investigate models of learning scenarios. The considered example systems, i.e., LVQ for classification and layered networks for regression, serve as examples to illustrate and demonstrate the usefulness of the methodology in obtaining principled insights into the properties of learning systems under concept drift. Typical behavior can be described in terms of learning curves, which reflect practically relevant phenomena such as the tracking of randomly varying targets or delayed learning in gradient descent due to quasi-stationary plateau states of the training process.

In the following sections, we first introduce the specific example systems, i.e., LVQ and SCM considered for classification and regression, respectively. In Section 2.3, we revisit the mathematical description of the learning dynamics in stationary environments for both systems. Next, the model is extended to include real concept drifts. We also briefly discuss the potential introduction of virtual drifts and the consideration of weight decay as an explicit mechanism of forgetting.

First results of our analysis are presented in Section 3, which exemplify and demonstrate the usefulness of the methodological approach: We obtain insights into the ability of prototype-based systems to track a time-varying classification scheme. Furthermore, we investigate the effect of concept drift on regression systems trained by gradient-based methods. In Section 4, we conclude with a general discussion and outlook on future work.

## 2. Models and Mathematical Analysis

We first introduce Learning Vector Quantization for classification with emphasis on the heuristic LVQ1 scheme. We further introduce a suitable, clustered density of input data, which is taken to define the target task in the model. Next, we present the Soft Committee Machine as an example regression system which can be studied in a so-called student–teacher scenario [5,6,7]. Here, training is based on stochastic gradient descent with respect to a suitable cost function.

In Section 2.3, we revisit the analytical treatment of on-line learning in stationary environments. We extend the mathematical framework with respect to the presence of concept drift in regression and classification in Section 2.4. In addition, we consider the incorporation of weight decay. Formally, the modifications compared to the stationary cases are identical in both scenarios.

### 2.1. Learning Vector Quantization

Learning Vector Quantization constitutes a family of prototype-based algorithms which are used in a wide variety of practical classification problems [26,27,28,29]. The popularity of the approach is due to several appealing properties: LVQ procedures are easy to implement and very intuitive. The classification of LVQ is based on a distance measure, frequently Euclidean, which is used to quantify the (dis-) similarity of feature vectors and class-specific prototypes. In contrast to the black-box character of many less transparent methods, LVQ allows for straightforward interpretations since the prototype vectors are embedded in the actual feature space and directly parameterize the classifier [28,29].

#### 2.1.1. Nearest Prototype Classification and Winner-Takes-All Training

In general, several prototypes can be employed to represent each class. In this contribution, however, we restrict the analysis to simple situations with only two prototypes ${\vec{w}}_{k}\in R^{N}$ in total, where prototype *k* is supposed to represent the data from Class k∈{1,2}.

A Nearest Prototype Classification (NPC) scheme is parameterized by the prototypes with respect to the distance measure $d(\vec{w},\vec{\xi })$: A given $\vec{\xi }\in R^{N}$ is assigned to the class of the closest prototype. In the presence of only two prototypes, the assignment is to Class 1 if $d\left({\vec{w}}_{1},\vec{\xi }\right)<d\left({\vec{w}}_{2},\vec{\xi }\right)$ and to Class 2, otherwise. In practice, ties can be broken arbitrarily.

A variety of distance measures can be used in LVQ, further enhancing the flexibility of the approach. Several popular choices, including adaptive distance measures in relevance learning, are discussed in [28,29,54]. In the following, we restrict ourselves to the most popular (squared) Euclidean measure

(1) $$d\left(\vec{w},\vec{\xi }\right)={\left(\vec{w}-\vec{\xi }\right)}^{2}.$$

We assume that, in the training process, a sequence of single example data $\left\{{\vec{\xi }}^{\;\mu },\sigma^{\mu }\right\}$ is presented to the system [8,9]. At time step μ=1,2,…, the vector ${\vec{\xi }}^{\;\mu }$ is presented, together with its class label $\sigma^{\mu }=1,2$. Generic incremental or on-line LVQ updates are of the form [39,46,47,48]:

(2) $${\vec{w}}_{k}^{\mu }\;={\vec{w}}_{k}^{\mu -1}\;+\Delta {\vec{w}}_{k}^{\mu }\;\;with\;\;\Delta {\vec{w}}_{k}^{\mu }\;=\;\frac{\eta }{N}\;f_{k}\left[d_{1}^{\mu },d_{2}^{\mu },\sigma^{\mu },\ldots \right]\;\left({\vec{\xi }}^{\mu }-{\vec{w}}_{k}^{\mu -1}\right)\;\;\mathrm{where}\;\;d_{i}^{\mu }=d\left({\vec{w}}_{i}^{\mu -1},{\vec{\xi }}^{\mu }\right)$$

and the learning rate η is scaled with the input dimension *N*. The precise algorithm is specified by choice of the modulation function $f_{k}\left[\ldots \right]$, which depends typically on the Euclidean distances of the data point from the current prototype positions and on the labels $k,\sigma^{\mu }=1,2$ of the prototype and training example.

Arguably the most basic LVQ training scheme was suggested by Kohonen and is known as LVQ1 [25,26,27]. In analogy to the NPC concept, it updates only the currently closest prototype according to a so-called Winner-Takes-All (WTA) scheme. Formally, the LVQ1 prescription for only two competing prototypes corresponds to Equation (2) with

(3) $$f_{k}\left[d_{1}^{\mu },d_{2}^{\mu },\sigma^{\mu }\right]\;=\Theta \left(d_{\hat{k}}^{\mu }-d_{k}^{\mu }\right)\Psi \left(k,\sigma^{\mu }\right),\;\;\mathrm{where}\;\;\hat{k}\;=\;\left\{\begin{array}{cc} 2 & \mathrm{if}\;k=1\\ 1 & \mathrm{if}\;k=2, \end{array}\right.$$

$$\Theta \left(x\right)\;=\;\left\{\begin{array}{cc} 1 & \mathrm{if}\;x>0\\ 0 & \mathrm{else}, \end{array}\right.\;\;\mathrm{and}\;\;\Psi \left(k,\sigma \right)=\;\left\{\begin{array}{cc} +1 & \mathrm{if}\;k=\sigma \\ -1 & \mathrm{else}. \end{array}\right.$$

Here, the Heaviside function Θ(…) singles out the winning prototype and the factor $\Psi (k,\sigma^{\mu })$ determines the sign of the update: The WTA update according to Equation (3) moves the prototype towards the presented feature vector if it carries the same class label $k=\sigma^{\mu }$. On the contrary, if the prototype is meant to present a different class, its distance from the data point is increased even further. Note that LVQ1 cannot be interpreted as a gradient descent procedure of a suitable cost function in a straightforward way due to discontinuities at the class boundaries.

Many modifications of LVQ have been suggested and discussed in the literature, including heuristically motivated extensions of LVQ1, cost function based schemes and variants employing unconventional or adaptive distance measures [25,26,27,28,29,54]. Mostly, they retain the basic idea of attraction and repulsion of the winning prototypes similar to Equation (3).

#### 2.1.2. Clustered Model Data

LVQ algorithms are most suitable for classification schemes which reflect a given cluster structure in the data. In the modeling, we therefore consider a stream of random input vectors $\vec{\xi }\in R^{N}$ which are generated independently according to a bi-modal distribution of the form [39,46,47,48]

(4) $$P\left(\vec{\xi }\right)=\sum_{m=1,2}\;p_{m}\;P\left(\vec{\xi }|m\right)\;\;\mathrm{with}\;\;P\left(\vec{\xi }|m\right)=\frac{1}{{\left(2\;\pi \;v_{m}\right)}^{N/2}}\;\exp\left[-\frac{1}{2\;v_{m}}{\left(\vec{\xi }-\lambda {\vec{B}}_{m}\right)}^{2}\right].$$

The target classification is taken to coincide with the cluster membership here, i.e., σ=m in Equation (3). Class-conditional densities $P(\vec{\xi }|m\;=\;1,2)$ correspond to isotropic, spherical Gaussians with variances $\;v_{m}$ and means $\lambda \;{\vec{B}}_{m}$. Prior weights of the clusters are denoted as $p_{m}$ and satisfy $p_{1}+p_{2}=1$. We assume that the vectors ${\vec{B}}_{m}$ are orthonormal with ${\vec{B}}_{1}^{\;2}={\vec{B}}_{2}^{\;2}=1$ and ${\vec{B}}_{1}\cdot {\vec{B}}_{2}=0$. Obviously, the classes m=1,2 are not linearly separable due to the overlap of the clusters.

**Figure 1:**
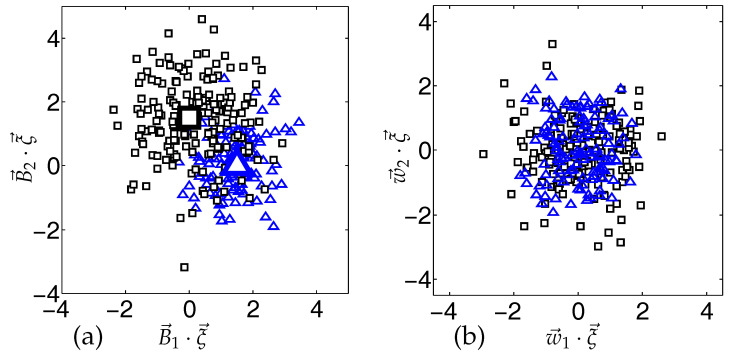
*Clustered Model Density*. Illustration of the clustered density, Equation (4), in N=200 dimensions, here with $p_{1}=0.4,\;p_{2}=0.6$ and $v_{1}=0.64,\;v_{2}=1.44$. Triangles (squares) represent 120(180) vectors $\vec{\xi }$ from the clusters centered at $\lambda {\vec{B}}_{1}$ ($\lambda {\vec{B}}_{2}$) with λ=1.5, respectively. (**a**) Projections ${\vec{B}}_{1,2}\cdot \vec{\xi }$ of the data. The cluster centers are marked by larger symbols. (**b**) Projections ${\vec{w}}_{1,2}\cdot \vec{\xi }$ on two randomly chosen orthonormal vectors ${\vec{w}}_{1,2}$.

As an illustration, Figure 1 displays data in N=200 dimensions, generated according to a density of the form in Equation (4). While the clusters are clearly visible in the subspace given by ${\vec{B}}_{1}$ and ${\vec{B}}_{2}$, projections into a randomly chosen plane completely overlap.

We denote conditional averages over $P(\vec{\xi }|m)$ as ${\langle \cdots \rangle }_{m}$, whereas mean values $\langle \cdots \rangle =\sum_{m=1,2}\;p_{m}\;{\langle \cdots \rangle }_{m}$ are defined with respect to the full density (Equation (4)). One obtains, for instance, the conditional and full averages

(5) $${\langle \vec{\xi }\rangle }_{m}=\lambda \;{\vec{B}}_{m},\;\;\;{\langle {\vec{\xi }}^{\;2}\rangle }_{m}=v_{m}\;N+\lambda^{2}\;\;\mathrm{and}\;\;\langle {\vec{\xi }}^{\;2}\rangle =\left(p_{1}v_{1}+p_{2}v_{2}\right)\;N+\lambda^{2}.$$

Note that, in the thermodynamic limit N→∞, which is considered below, $\lambda^{2}$ can be neglected in comparison to the terms of O(N) in Equation (5).

### 2.2. Soft Committee Machines

The term Soft Committee Machine (SCM) has been coined for feedforward neural networks with sigmoidal activations in a single hidden layer and a linear output unit (see, for instance, [30,31,32,33,34,35,36,55,56]). Its structure resembles that of a (crisp) committee machine with binary threshold hidden units, where the network’s response is given by their majority vote (see [5,6,7] and references therein).

#### 2.2.1. Network Definition

The output of an SCM with *K* hidden units and fixed hidden-to-output weights is of the form

(6) $$y\left(\vec{\xi }\right)=\sum_{k=1}^{K}\;g\left({\vec{w}}_{k}\cdot \vec{\xi }\right)$$

where ${\vec{w}}_{k}\in R^{N}$ denotes the weight vector connecting the *N*-dimensional input layer with the *k*th hidden unit. A non-linear transfer function g(⋯) defines the hidden unit states and the final output is given as their sum. As a specific example, we consider the sigmoidal

(7) $$g\left(x\right)=\mathrm{erf}\left(x/\sqrt{2}\right)\;\;\mathrm{with}\;\mathrm{the}\;\mathrm{derivative}\;\;g^{\prime }\left(x\right)=\sqrt{\frac{2}{\pi }}\;e^{-x^{2}/2}$$

The activation resembles closely other sigmoidal functions, e.g., the popular tanh(x), but offers great mathematical ease in the analytical treatment, as originally exploited in [30].

Note that the SCM, cf. Equation (6), is not quite representing a universal approximator, a property which could be achieved by introducing adaptive local thresholds $\vartheta_{i}\in R$ in hidden unit activations of the form $g\left({\vec{w}}_{i}\cdot \vec{\xi }-\vartheta_{i}\right)$ (see [57] for a general proof). Adaptive hidden-to-output weights also increase the flexibility of the SCM and have been studied in, for instance [33], from a statistical physics perspective. Here, however, the emphasis is on basic dynamical effects in the on-line training of an SCM and we restrict ourselves to the simpler model defined above.

#### 2.2.2. Regression Scheme and On-Line Gradient Descent

In the context of continuous regression, the training of neural networks with output $y(\vec{\xi })$ based on examples $\left\{{\vec{\xi }}^{\mu }\in R^{N},\tau^{\mu }\in R\right\}$ is frequently guided by the quadratic deviation of the network output from the target values [1,2,3]. It serves as a cost function which evaluates the network performance with respect to a single example as

(8) $$e^{\mu }\left({\left\{{\vec{w}}_{k}\right\}}_{k=1}^{K}\right)=\frac{1}{2}{\left(y^{\mu }-\tau^{\mu }\right)}^{2}\;\;\;\mathrm{with}\;\mathrm{the}\;\mathrm{shorthand}\;\;y^{\mu }=y\left({\vec{\xi }}^{\mu }\right).$$

In stochastic or on-line gradient descent, updates of the weight vectors are based on the sequential presentation of single examples:

(9) $${\vec{w}}_{k}^{\mu }={\vec{w}}_{k}^{\mu -1}+\Delta {\vec{w}}_{k}^{\mu }\;\;\mathrm{with}\;\;\;\Delta {\vec{w}}_{k}^{\mu }=\;-\frac{\eta }{N}\;\frac{\partial e^{\mu }}{\partial {\vec{w}}_{k}}e^{\mu }\;=\;-\frac{\eta }{N}\left(y^{\mu }-\tau^{\mu }\right)\frac{\partial }{\partial {\vec{w}}_{k}}y^{\mu }$$

where the gradient is evaluated in ${\vec{w}}_{k}^{\mu -1}$. For the SCM architecture specified above, we have

(10) $$\frac{\partial y^{\mu }}{\partial {\vec{w}}_{k}}=g^{\prime }\left(h_{k}^{\mu }\right){\vec{\xi }}^{\mu }\;\;\mathrm{and}\;\;\Delta {\vec{w}}_{k}^{\mu }=-\frac{\eta }{N}\left(\sum_{i=1}^{K}\mathrm{erf}\left[\frac{1}{\sqrt{2}}h_{i}^{\mu }\right]-\tau^{\mu }\right)\;\frac{1}{\sqrt{2\pi }}\exp\left[-\frac{1}{2}{\left(h_{k}^{\mu }\right)}^{2}\right]\;{\vec{\xi }}^{\mu }$$

with the inner products $h_{i}^{\mu }={\vec{w}}_{i}^{\mu -1}\cdot {\vec{\xi }}^{\mu }$ of the current weight vectors with the new example input. Note that the change of weight vectors is proportional to ${\vec{\xi }}^{\mu }$ and can be seen as a form of Hebbian Learning [1,2,3].

#### 2.2.3. Student–Teacher Scenario and Model Data

To define and model meaningful learning situations, we resort to the consideration of student–teacher scenarios [5,6,7,8]. We assume that the target regression can be defined in terms of an SCM with a given number *M* of hidden units and a specific set of weights ${\left\{{\vec{B}}_{m}\in R^{N}\right\}}_{m=1}^{M}$:

(11) $$\tau \left(\vec{\xi }\right)=\sum_{m=1}^{M}\;g\left({\vec{B}}_{m}\cdot \vec{\xi }\right).$$

In the model, this so-called teacher network can be equipped with M>K hidden units to model regression schemes which cannot be learnt by an SCM student of the form in Equation (6). On the contrary, K>M would correspond to an over-learnable target. For the discussion of these highly interesting cases in stationary environments, see, for instance, [30,31,32,33,34]. In a student-teacher scenario with *K* and *M* hidden units, respectively, the update of the student weight vectors by on-line gradient descent reads:

(12) $${\vec{w}}_{k}^{\mu }={\vec{w}}_{k}^{\mu -1}-\frac{\eta }{N}\;\rho_{k}^{\mu }\;{\vec{\xi }}^{\mu }\;\;\mathrm{where}\;\;\rho_{k}^{\mu }=\;\left(\sum_{i=1}^{K}\mathrm{erf}\left[\frac{h_{i}^{\mu }}{\sqrt{2}}\right]-\sum_{m=1}^{M}\mathrm{erf}\left[\frac{b_{m}^{\mu }}{\sqrt{2}}\right]\right)\;\frac{1}{\sqrt{2\pi }}\exp\left[-\frac{1}{2}{\left(h_{k}^{\mu }\right)}^{2}\right]$$

with the quantities $b_{m}^{\mu }={\vec{B}}_{m}\cdot {\vec{\xi }}^{\mu }$ and $h_{k}^{\mu }={\vec{w}}_{k}^{\mu -1}\cdot {\vec{\xi }}^{\mu }$.

In the following, we restrict our analysis to perfectly matching student complexity with K=M=2 only, which further simplifies Equation (12). Extensions to more hidden units and settings with K≠M will be considered in forthcoming projects.

In contrast to the model for LVQ-based classification, the vectors ${\vec{B}}_{m}$ define the target output $\tau^{\mu }=\tau \left({\vec{\xi }}^{\mu }\right)$ explicitly via the teacher network for any input vector. While clustered input densities of the form in Equation (4) can also be studied for feedforward networks as in [44,45], we assume here that the actual input vectors are uncorrelated with the teacher vectors ${\vec{B}}_{m}$. Consequently, we can resort to a simpler model density and consider vectors $\vec{\xi }$ of independent, zero mean, unit variance components with, e.g.,

(13) $$P\left(\vec{\xi }\right)=\frac{1}{{\left(2\;\pi \right)}^{N/2}}\;\exp\left[-\frac{1}{2}{\left(\;\vec{\xi }\;\right)}^{2}\right].$$

Note that Equation (13) could be recovered formally from the density (Equation (4)) as a special case with parameters λ=0 and $v_{1}=v_{2}=1$, for which the two clusters coincide around the origin and $p_{1,2}$ become irrelevant.

### 2.3. The Dynamics of On-Line Training in Stationary Environments

In the following, we sketch the successful theory of on-line learning [5,6,7,8,38] as, for instance, applied to the dynamics of LVQ algorithms in [39,46,47,48] and to on-line gradient descent in SCM in [30,31,32,33,34,35,36]. We refer the reader to the original publications for details. The extensions to non-stationary situations with concept drifts are discussed in Section 2.4.

The analysis follows the same key steps in both settings. We consider adaptive vectors ${\vec{w}}_{1,2}\in R^{N}$ (prototypes in LVQ or student weights in the SCM) while the characteristic vectors ${\vec{B}}_{1,2}$ specify the target task (cluster centers in LVQ training, SCM teacher vectors for regression).

The consideration of the thermodynamic limit N→∞ is instrumental for the theoretical treatment. The limit facilitates the following key steps, which, eventually, yield an exact mathematical description of the training dynamics in terms of ordinary differential equations (ODE):

(a) *Order parameters*

The many degrees of freedom, i.e., the components of the adaptive vectors, can be characterized in terms of only very few quantities. The definition of meaningful so-called *order parameters* follows naturally from the specific mathematical structure of the model. After presentation of a number μ of examples, as indicated by corresponding superscripts, we describe the system by the projections

(14) $$R_{im}^{\mu }\;=\;{\vec{w}}_{i}^{\mu }\;\cdot {\vec{B}}_{m}\;\mathrm{and}\;\;Q_{ik}^{\mu }\;=\;{\vec{w}}_{i}^{\mu }\;\cdot {\vec{w}}_{k}^{\mu }\;\mathrm{with}\;\;\;i,k,m\in \left\{1,2\right\}.$$

Obviously, $Q_{11}^{\mu },Q_{22}^{\mu }$ and $Q_{12}^{\mu }=Q_{21}^{\mu }$ relate to the norms and mutual overlap of the adaptive vectors, while the four quantities $R_{im}$ specify their projections into the linear subspace defined by the characteristic vectors $\left\{{\vec{B}}_{1},{\vec{B}}_{2}\right\}$, respectively.

(b) Recursions

For the order parameters, recursion relations can be derived directly from the learning algorithms in Equations (2) and (9), which are both of the generic form ${\vec{w}}_{k}^{\mu }\;={\vec{w}}_{k}^{\mu -1}\;+\Delta {\vec{w}}_{k}^{\mu },$ by considering the corresponding inner products:

(15) $$\begin{array}{ccc} \frac{R_{im}^{\mu }-R_{im}^{\mu -1}}{1/N} & = & \eta \;\Delta {\vec{w}}_{i}^{\mu }\cdot {\vec{B}}_{m}\;\\ \frac{Q_{ik}^{\mu }-Q_{ik}^{\mu -1}}{1/N} & = & \eta \left({\vec{w}}_{i}^{\mu -1}\cdot \Delta {\vec{w}}_{k}^{\mu }+{\vec{w}}_{k}^{\mu -1}\cdot \Delta {\vec{w}}_{i}^{\mu }\right)+\;\eta^{2}\;\Delta {\vec{w}}_{i}^{\mu }\cdot \Delta {\vec{w}}_{k}^{\mu }. \end{array}$$

Note that terms of order O(1/N) on the right hand side (r.h.s.) of Equation (15) will be neglected in the following.

(c) Averages over the Model Data

Applying the central limit theorem (CLT), we can perform an average over the random sequence of independent examples. Note that $\Delta {\vec{w}}_{k}^{\mu }\propto {\vec{\xi }}^{\mu }$ or $\Delta {\vec{w}}_{k}^{\mu }\propto \left({\vec{\xi }}^{\mu }-{\vec{w}}_{k}^{\mu -1}\right)$, respectively.

Consequently, the current input ${\vec{\xi }}^{\mu }$ enters the r.h.s. of Equation (15) only through its norm $|\vec{\xi }{|}^{2}=O\left(N\right)$ and the quantities

(16) $$h_{i}^{\mu }\;={\vec{w}}_{i}^{\mu -1}\cdot {\vec{\xi }}^{\mu }\;\;\mathrm{and}\;\;b_{m}^{\mu }\;={\vec{B}}_{m}\cdot {\vec{\xi }}^{\mu }.$$

Since these inner products correspond to sums of many independent random quantities in our model, the CLT implies that the projections in Equation (16) are correlated Gaussian quantities for large *N* and their joint density $P(h_{1}^{\mu },h_{2}^{\mu },b_{1}^{\mu },b_{2}^{\mu })$ is given completely by first and second moments.

*LVQ:* For the clustered density, cf. Equation (4), the conditional moments read

$${\langle h_{i}^{\mu }\rangle }_{m}=\lambda R_{im}^{\mu -1},\;{\langle b_{m}^{\mu }\rangle }_{n}=\lambda \delta_{mn},\;{\langle h_{i}^{\mu }h_{k}^{\mu }\rangle }_{m}-{\langle h_{i}^{\mu }\rangle }_{m}{\langle h_{k}^{\mu }\rangle }_{m}=v_{m}\;Q_{ik}^{\mu -1},$$

(17) $$\;{\langle h_{i}^{\mu }b_{n}^{\mu }\rangle }_{m}-{\langle h_{i}^{\mu }\rangle }_{m}{\langle b_{n}^{\mu }\rangle }_{m}=v_{m}\;R_{in}^{\mu -1},\;{\langle b_{l}^{\mu }b_{n}^{\mu }\rangle }_{m}-{\langle b_{l}^{\mu }\rangle }_{m}{\langle b_{n}^{\mu }\rangle }_{m}=v_{m}\;\delta_{ln},$$

where i,k,l,m,n∈{1,2} and $\delta_{\ldots }$ is the Kronecker-Delta.

*SCM:* In the simpler case of the isotropic, spherical density (Equation (13)) with λ=0 and $v_{1}=v_{2}=1$ the moments reduce to

$$\langle h_{i}^{\mu }\rangle =0,\;\langle b_{m}^{\mu }\rangle =0,\;\langle h_{i}^{\mu }h_{k}^{\mu }\rangle -\langle h_{i}^{\mu }\rangle \langle h_{k}^{\mu }\rangle =Q_{ik}^{\mu -1}$$

(18) $$\;\langle h_{i}^{\mu }b_{n}^{\mu }\rangle -\langle h_{i}^{\mu }\rangle \langle b_{n}^{\mu }\rangle =\;R_{in}^{\mu -1},\;\langle b_{l}^{\mu }b_{n}^{\mu }\rangle -\langle b_{l}^{\mu }\rangle \langle b_{n}^{\mu }\rangle =\delta_{ln}.$$

Hence, in both cases, the joint density of $h_{1,2}^{\mu }$ and $b_{1,2}^{\mu }$ is fully specified by the values of the order parameters in the previous time step and the parameters of the model density. This important result enables us to perform an average of the recursion relations (Equation (15)) over the latest training example in terms of Gaussian integrals. Moreover, the resulting r.h.s. can be expressed in closed form in $\left\{R_{im}^{\mu -1},Q_{ik}^{\mu -1}\right\}$.

(d) *Self-Averaging Properties*

The self-averaging property of order parameters makes it possible to restrict the description to their mean values: Fluctuations of the stochastic dynamics can be neglected in the limit N→∞. This concept has been borrowed from the statistical physics of disordered materials and has been applied frequently in the study of neural network models and learning processes [4,5,6,7]. For a detailed mathematical discussion in the context of sequential on-line learning, see [58].

Consequently, we can interpret the averaged Equation (15) directly as deterministic recursions for the means of $\left\{R_{im}^{\mu },Q_{ik}^{\mu }\right\}$ which coincide with their actual values in the thermodynamic limit.

(e) *Continuous Time Limit and ODE*

For N→∞, we can interpret the ratios on the left hand sides of Equation (15) as derivatives with respect to the continuous learning time

(19) α=μ/N.

This scaling corresponds to the plausible assumption that the number of examples required for successful training is proportional to the number of degrees of freedom in the system.

Averages are performed over the joint densities $P\left(\left\{h_{i},b_{m}\right\}\right)$ corresponding to the most recent, independently drawn input vector. Here, and in the following, we have omitted the index μ.

The resulting sets of coupled ODE obtained from Equation (15) are of the generic form:

(20) $${\left[\frac{dR_{im}}{d\alpha }\right]}_{stat}=\eta F_{im}\;\;\;\mathrm{and}\;\;\;{\left[\frac{dQ_{ik}}{d\alpha }\right]}_{stat}\;\;\;=\eta G_{ik}^{\left(1\right)}+\eta^{2}G_{ik}^{\left(2\right)}.$$

Here, the subscript *stat* indicates that the ODE describe learning from a stationary density, cf. Equation (4) or (13).

*LVQ:* For the classification model, we have to insert the terms

$$F_{im}=\left(\langle b_{m}f_{i}\rangle \;-\;R_{im}\langle f_{i}\rangle \right),\;$$

(21) $$G_{ik}^{\left(1\right)}=\left(\langle h_{i}f_{k}+h_{k}f_{i}\rangle \;-\;Q_{ik}\langle f_{i}\;+\;f_{k}\rangle \right)\;\;\mathrm{and}\;\;G_{ik}^{\left(2\right)}=\sum_{m=1,2}v_{m}p_{m}{\langle f_{i}f_{k}\rangle }_{m}$$

with the LVQ1 modulation functions $f_{i}$ from Equation (3) and (conditional) averages with respect to the density (Equation (4)).

*SCM:* In the modeling of regression in a student–teacher scenario, we obtain

(22) $$F_{im}=\langle \rho_{i}b_{m}\rangle ,\;G_{ik}^{\left(1\right)}=\langle \left(\rho_{i}h_{k}+\rho_{k}h_{i}\right)\rangle \;\;\mathrm{and}\;\;G_{ik}^{\left(2\right)}=\langle \rho_{i}\rho_{k}\rangle$$

where the quantities $\rho_{i}$ are defined in Equation (12) for the latest input vector and averages are performed over the isotropic input density (Equation (13)).

In both training scenarios considered here, the r.h.s. of Equation (20), as given by Equations (21) and (22), can be expressed in terms of elementary functions. For the straightforward yet lengthy results, we refer the reader to the original literature for LVQ [39,46] and SCM [31,32,33,34].

(f) Generalization error

After training, the success of learning is quantified in terms of the generalization error $\epsilon_{g}$, which can also be expressed as a function of order parameters.

*LVQ:* In the case of classification, $\epsilon_{g}$ is given as the probability of misclassifying a novel, randomly drawn input vector. In the LVQ model, class-specific errors corresponding to data from clusters k=1,2 in Equation (4) can be considered separately:

(23) $$\epsilon_{g}\;=\;p_{1}\;\epsilon_{g}^{1}\;+\;p_{2}\;\epsilon_{g}^{2},\;\;\;\mathrm{where}\;\;\epsilon_{g}^{k}\;=\;{\langle \Theta \left(d_{k}-d_{\hat{k}}\right)\rangle }_{k}$$

is the class-specific misclassification rate, i.e., the probability for an example drawn from a cluster *k* to be assigned to $\hat{k}\neq k$ with $d_{k}>d_{\hat{k}}$. For the derivation of the class-wise and total generalization error for systems with two prototypes as functions of the order parameters, we also refer to [39]. One obtains

(24) $$\epsilon_{g}^{k}\;=\;\Phi \left(\frac{Q_{kk}-Q_{\hat{k}\hat{k}}-2\lambda \left(R_{kk}-R_{\hat{k}k}\right)}{2\sqrt{v_{k}}\sqrt{Q_{11}-2Q_{12}+Q_{22}}}\right)\;\;\mathrm{where}\;\;\Phi \left(z\right)=\int_{-\infty }^{z}dx\frac{e^{-x^{2}/2}}{\sqrt{2\pi }}.$$

*SCM:* For regression, the generalization error is defined as an average $\langle \cdots \rangle$ of the quadratic deviation between student and teacher output over the isotropic density, cf. Equation (13):

(25) $$\epsilon_{g}\;=\frac{1}{2}\langle {\left(\sum_{k=1}^{K}\mathrm{erf}\left[\frac{h_{k}}{\sqrt{2}}\right]-\sum_{m=1}^{M}\mathrm{erf}\left[\frac{b_{m}}{\sqrt{2}}\right]\right)}^{2}\rangle ,$$

the full form of which can be found in [31,32] for arbitrary *K* and *M*. For K=M=2 with orthonormal teacher vectors, it simplifies to

(26) $$\epsilon_{g}\;=\frac{1}{3}+\frac{1}{\pi }\left[\sum_{i,k=1}^{2}\arcsin\left(\frac{Q_{ik}}{\sqrt{1+Q_{ii}}\sqrt{1+Q_{kk}}}\right)-2\sum_{i,m=1}^{2}\arcsin\left(\frac{R_{im}}{\sqrt{2}\sqrt{1+Q_{ii}}}\right)\right].$$

(g) *Learning curves*

The (numerical) integration of the ODE for a given particular training algorithm, model density and specific initial conditions, $\left\{R_{im}\left(0\right),Q_{ik}\left(0\right)\right\}$ yields the temporal evolution of order parameters in the course of training.

Exploiting the self-averaging properties of order parameters once more, we can obtain the learning curves $\epsilon_{g}\left(\alpha \right)=\epsilon_{g}\left(\{R_{im}\left(\alpha \right),Q_{ik}\left(\alpha \right)\}\right)$, i.e., the generalization error after on-line training with (αN) random examples.

### 2.4. The Learning Dynamics Under Concept Drift

The analysis summarized in the previous section concerns learning in the presence of a stationary concept, i.e., for a density of the form of Equation (4) or (13) with characteristic vectors ${\vec{B}}_{1,2}$ which do not change in the course of training. Here, we discuss the effect of concept drift on the learning process within the modeling framework and consider weight decay as an explicit mechanism of forgetting.

#### 2.4.1. Virtual Drift

Several virtual drift processes can be studied in appropriate modifications of the basic framework. Virtual drifts affect the statistical properties of observed example data, while the actual target task remains the same. As one example, time-varying label noise could be incorporated into both models in a straightforward way [5,6,7]. Similarly, non-stationary cluster variances in the input density, cf. Equation (4), can be considered by assuming explicitly time-dependent $v_{\sigma }\left(\alpha \right)$ in Equation (20). A particularly relevant case would be that of non-stationary prior probabilities $p_{\sigma }\left(\alpha \right)$ in classification, where a varying fraction of examples represents each of the classes in the data stream. In practical situations, varying class bias can complicate the training significantly and lead to inferior performance.

We will investigate these and similar, purely virtual drift processes in forthcoming studies.

#### 2.4.2. Real Drift

In the presented framework, a real drift can be modeled as a process which displaces the characteristic vectors ${\vec{B}}_{1,2}$ (cluster centers in LVQ, teacher weight vectors in the SCM) in the *N*-dimensional feature space. Various scenarios could be considered; we restrict ourselves to the analysis of a random diffusion of vectors ${\vec{B}}_{1,2}\left(\mu \right)$. Upon presentation of example μ, we assume that random vectors ${\vec{B}}_{1,2}\left(\mu \right)$ are generated which satisfy the conditions

$${\vec{B}}_{1}\left(\mu \right)\cdot {\vec{B}}_{1}\left(\mu -1\right)={\vec{B}}_{2}\left(\mu \right)\cdot {\vec{B}}_{2}\left(\mu -1\right)=\left(1-\frac{\delta }{N}\right)$$

(27) $${\vec{B}}_{1}\left(\mu \right)\cdot {\vec{B}}_{2}\left(\mu \right)=0\;\mathrm{and}\;|{\vec{B}}_{1}\left(\mu \right){|}^{2}=|{\vec{B}}_{2}\left(\mu \right){|}^{2}=1.$$

Here, δ quantifies the strength of the drift process. The displacement of the characteristic vectors is very small in an individual training step and we assume for simplicity that orthonormality is preserved. In terms of the above defined continuous time α=μ/N, the drift parameter sets the time scale 1/δ on which the vectors lose memory of their previous positions according to ${\vec{B}}_{m}\left(\alpha_{1}\right)\cdot {\vec{B}}_{m}\left(\alpha_{o}\right)\;=\exp\left[-\delta \left(\alpha_{1}-\alpha_{o}\right)\right]\;\;\mathrm{for}\;\alpha_{1}>\alpha_{o}.$

The effect of such a drift process can be accounted for in the mathematical analysis of the dynamics in a straightforward way: For a given vector ${\vec{w}}_{i}\in R^{N}$, we obtain [50,51,52,53]

(28) $$\left[{\vec{w}}_{i}\cdot {\vec{B}}_{k}\left(\mu \right)\right]=\left(1-\frac{\delta }{N}\right)\;\left[{\vec{w}}_{i}\cdot {\vec{B}}_{k}\left(\mu -1\right)\right]\;\;\mathrm{for}\;k=1,2$$

under the above specified small displacement in discrete learning time. Hence, the drift tends to decrease the student–teacher overlaps continuously which clearly deteriorates the success of training compared with the stationary case. The resulting ODE for the training dynamics in the limit N→∞ under the drift process (Equation (27)) reads

(29) $${\left[\frac{dR_{im}}{d\alpha }\right]}_{drift}\;=\;{\left[\frac{dR_{im}}{d\alpha }\right]}_{stat}\;-\delta \;R_{im}\;\;\;\mathrm{and}\;\;\;{\left[\frac{dQ_{ik}}{d\alpha }\right]}_{drift}={\left[\frac{dQ_{ik}}{d\alpha }\right]}_{stat}$$

with the terms ${\left[\cdots \right]}_{stat}$ for stationary environments taken from Equation (20). However, as the teacher vectors are time-dependent, order parameters $R_{im}\left(\alpha \right)$ correspond to the inner products ${\vec{w}}_{i}^{\mu }\cdot {\vec{B}}_{m}\left(\mu \right)$, here.

#### 2.4.3. Weight Decay

Possible motivations for the introduction of so-called weight decay in machine learning systems range from regularization as to reduce the risk of over-fitting in regression and classification [1,2,3] to the modeling of forgetful memories in attractor neural networks [59,60].

Here, we introduce weight decay as an element of explicit forgetting to potentially improve the performance of the trained systems in the presence of real concept drift. To this end, we consider the multiplication of all adaptive vectors by a factor (1-γ/N) before the generic learning step given by $\Delta {\vec{w}}_{i}^{\mu }$ in Equation (2) or (9), respectively:

(30) $${\vec{w}}_{i}^{\mu }\;=\;\left(1-\frac{\gamma }{N}\right)\;{\vec{w}}_{i}^{\mu -1}\;+\Delta {\vec{w}}_{i}^{\mu }.$$

Analogous modifications of perceptron training under concept drift were discussed in [50,51,52,53], and weight decay in the SCM has been studied in [61,62]. Since the multiplications with $\left(1-\gamma /N\right)$ accumulate in the course of training, weight decay enforces an increased influence of the most recent training data as compared to earlier examples.

In the thermodynamic limit N→∞, the modified ODE for training under real drift, cf. Equation (27), and weight decay, Equation (30), are obtained in a straightforward manner and read

(31) $${\left[\frac{dR_{im}}{d\alpha }\right]}_{decay}\;\;\;\;=\;{\left[\frac{dR_{im}}{d\alpha }\right]}_{stat}\;\;\;\;\;-\left(\delta +\gamma \right)R_{im}\;\;\;\mathrm{and}\;\;\;{\left[\frac{dQ_{ik}}{d\alpha }\right]}_{decay}\;\;\;\;=\;{\left[\frac{dQ_{ik}}{d\alpha }\right]}_{stat}\;\;\;\;\;-2\;\gamma \;Q_{ik}$$

with the terms for stationary environments in absence of weight decay, Equation (20).

## 3. Results and Discussion

We present and discuss first results that illustrate the usefulness of the modeling framework. First, we obtain insight into the capability of LVQ to cope with concept drift in classification. Second, we investigate the non-trivial effects of drift on the on-line gradient descent training of layered neural networks in regression tasks.

### 3.1. Learning Vector Quantization in the Presence of Real Concept Drift

We study the typical behavior of LVQ1 under real concept drift as defined in Section 2.4.2. Throughout the following, we consider prototypes initialized as independent, normalized random vectors with no prior knowledge of the cluster structure, which corresponds to

(32) $$Q_{11}\left(0\right)=Q_{22}\left(0\right)=1,\;\;Q_{12}\left(0\right)=0\;\;\mathrm{and}\;\;R_{im}\left(0\right)=0\;\;\mathrm{for}\;\;i,m\in \left\{1,2\right\}.$$

**Figure 2:**
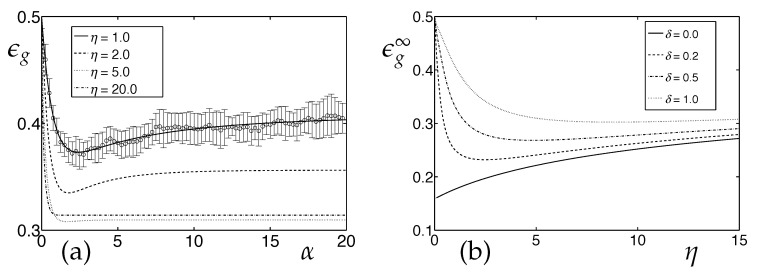
*LVQ under Concept Drift: Learning Curves and the Role of the Learning Rate*. LVQ1 training from data according to the model density (Equation (4)) with $\lambda =1,p_{1}=p_{2}=0.5$ and $v_{1}=v_{2}=0.5$ in the presence of real concept drift. (**a**) Learning curves $\epsilon_{g}\left(\alpha \right)$ for δ=1 and various learning rates η. Symbols and error bars mark the mean results and standard deviations observed in 25 randomized simulations for N=1000 with η=1 as an example. (**b**) Asymptotic (α→∞) generalization error as a function of the learning rate η for different drift parameters δ and in the stationary environment with δ=0.

Figure 2a displays example learning curves $\epsilon_{g}\left(\alpha \right)$ for a drift with δ=1 for different learning rates, see the caption for other model parameters. Details of the initial phase of training, depend on the interplay of initial values $Q_{ii}\left(0\right)$ and the learning rate. Note that a non-monotonic behavior of $\epsilon_{g}\left(\alpha \right)$ can be observed for some settings.

Monte Carlo simulations show excellent agreement with the (N→∞) theoretical predictions already for relatively small systems. This parallels the findings presented in [39,46] for stationary environments. As just one example, Figure 2a also shows the mean and standard deviation of $\epsilon_{g}$ over 25 randomized runs of the training for η=1 and N=1000. A systematic comparison and discussion of the *N*-dependence in computer experiments of LVQ under concept drift will be presented elsewhere.

The results for large α show that the success of learning, i.e., the degree to which the drifting concept can be tracked by LVQ1, depends on the learning rate in a non-trivial way. In contrast to learning in stationary environments, the use of very small learning rates obviously fails to maintain the ability to generalize in the presence of a significant real drift. On the other hand, too large learning rates result in inferior performance as well.

After presenting many examples, i.e., in the limit α→∞, the system approaches a quasi-stationary state in which the LVQ prototypes track the drifting center vectors ${\vec{B}}_{1,2}$ with constant overlap parameters $R_{im},Q_{ik}$. The configuration corresponds to the stationarity conditions

(33) $${\left[\frac{dR_{im}}{d\alpha }\right]}_{drift}\;\;\;\;=\;0\;\;\;\mathrm{and}\;\;\;{\left[\frac{dQ_{ik}}{d\alpha }\right]}_{drift}\;\;\;\;=\;0.$$

Figure 2b shows the α→∞ asymptotic generalization error $\epsilon_{g}^{\infty }=\lim_{\alpha \to \infty }\epsilon_{g}\left(\alpha \right)$ as a function of η. Only in absence of drift, i.e., for δ=0, the best possible generalization ability of LVQ1 is obtained in the limit η→0. We refer the reader to [39,46] for a detailed discussion of $\epsilon_{g}^{\infty }$ and its dependence of the model parameters $\lambda ,p_{\pm }$ and $v_{\pm }$. For δ>0, the limit η→0 results in trivial asymptotic behavior corresponding to random guesses, with $\epsilon_{g}^{\infty }=1/2$ for the symmetric input density with $p_{1}=p_{2}$ and $v_{1}=v_{2}$, for instance.

**Figure 3:**
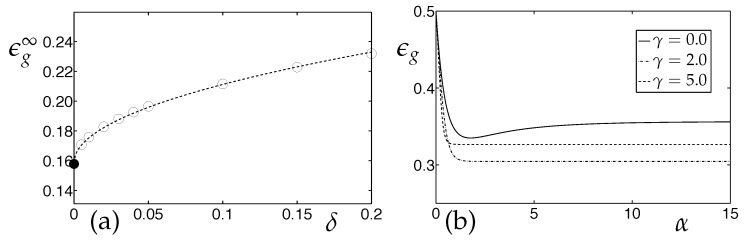
*LVQ under Concept Drift: Asympotic Generalization and the Influence of Weight Decay*. LVQ1 in the presence of a real drift with model parameters $\lambda =1,v_{1}=v_{2}=0.5,p_{1}=p_{2}=0.5$. (**a**) The (α→∞) asymptotic generalization error of LVQ1 as obtained with an optimized constant learning rate. Empty circles correspond to numerical results for different drift parameters, the filled circle represents stationary data, for which $\epsilon_{g}^{\infty }\left(\delta \;=\;0\right)\approx 0.158$. The dashed line corresponds to a fit of the form $\epsilon_{g}^{\infty }\left(\delta \;=\;0\right)+0.166\;\delta^{1/2}$. (**b**) Learning curves in the model with learning rate η=2.0 and drift parameter δ=1.0. The three curves correspond to learning without weight decay (upper, solid line), with γ=2 (lower, dash-dotted line) and γ=5 (middle, dashed line).

Given the drift parameter δ, an optimal constant learning rate can be identified with respect to the generalization ability in the quasi-stationary state. The use of this learning rate yields, for α→∞, the best $\epsilon_{g}^{\infty }$ achievable under drift. It is displayed in Figure 3a as a function of δ for small values of the drift parameter. The optimal quasi-stationary generalization error under concept drift scales is:

(34) $$\left[\epsilon_{g}^{\infty }\left(\delta \right)-\epsilon_{g}^{\infty }\left(0\right)\right]\;\propto \delta^{1/2}\;\;\mathrm{for}\;\mathrm{small}\;\;\delta .$$

As expected, the drift impedes the learning process. However, our results show that already the simplest LVQ scheme is capable of tracking randomly drifting clusters and to maintain a significant generalization ability, even in very high-dimensional spaces.

We have also studied the effect of weight decay in the presence of the above discussed real concept drift. Figure 3b displays example learning curves for LVQ1 training with various weight decay parameters γ for a given learning rate η. As these examples show, the implementation of weight decay has the potential to improve the generalization behavior significantly when tracking a drifting concept. The simultaneous optimization of learning rate and weight decay {η,γ} with respect to the success of training in the tracking state will be addressed in forthcoming studies.

### 3.2. SCM Regression in the Presence of Real Concept Drift

Here, we present results concerning the SCM student-teacher scenario with K=M=2. Already in this simplest setting and in absence of concept drift, the learning dynamics displays non-trivial phenomena which have been studied in detail in, among others, [31,32,34]. Perhaps the most interesting effect is the occurrence of quasi-stationary plateau-states which can even dominate the learning curves $\epsilon_{g}\left(\alpha \right)$. They reflect the existence of weakly repulsive fixed points of the ODE (Equation (20)) and correspond to sub-optimal, more or less symmetric configurations of the student network. The problem of delayed learning due to saddle points and related effects in gradient-based training is obviously also of interest in the context of Deep Learning (see [3,37,63,64] for recent investigations and further references).

In the SCM model, one can show that a plateau with $R_{ik}\approx R$ and and $Q_{ik}\approx Q\;\;forall\;\;i,k\in \left\{1,2\right\}$ always exists in the case of orthonormal teacher vectors and for small learning rates [31,32,34]. In this state, all student weight vectors have acquired the same, limited knowledge of the target rule. To achieve better generalization ability, this symmetry has to be broken or, in other words, the student hidden units have to specialize and represent specific units of the teacher network.

Note that more complex fixed point configurations with different degrees of (partial) specialization can be found, in general. The number of observable plateaus depends on the learning rate and increases for larger *K* and *M* (see [34] for a detailed discussion in the absence of drift).

In practice, one expects $R_{im}\left(0\right)\approx 0$ for all i,m unless prior knowledge is available about the target. Hence, the student specialization $S_{i}\left(0\right)=\left|R_{i1}\left(0\right)-R_{i2}\left(0\right)\right|$ is also expected to be small, initially. A nearly unspecialized configuration with $S_{i}\left(\alpha \right)\approx 0$ persists in a transient phase of learning, which can extend over large values of α. The actual shape and length of the plateau depends on the precise initialization and the repulsive properties of the corresponding fixed point of the dynamics (see [34] for a detailed discussion, which also addresses the effect of finite *N* in Monte Carlo simulations).

**Figure 4:**
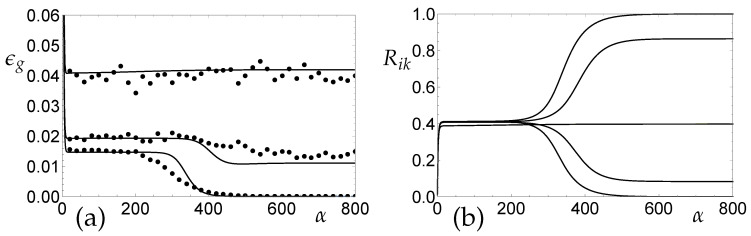
*Regression under Concept Drift: Learning Curves*. Gradient-based training of the Soft Committee Machine with K=M=2 and orthogonal teacher vectors in the presence of real target drift, with learning rate η=0.5 and initial conditions as specified in Equation (35). (**a**) Learning curves for the stationary case with δ=0 (lower line), for weak drift with δ=0.005 (middle) and for strong drift with δ=0.03 (upper line). Symbols represent the result of single Monte Carlo simulation runs for system size N=500. (**b**) The corresponding evolution of the student–teacher overlaps $R_{11}=R_{22}$ and $R_{12}=R_{21}$ vs. α for the stationary case with δ=0 (lower and upper lines), for weak drift with δ=0.005 (intermediate) and strong drift with δ=0.03 (center, all overlaps equal).

Figure 4a shows an example (lowest curve) of a pronounced plateau state in on-line gradient descent for initial conditions

(35) $$R_{im}=R_{o}+U\left(10^{-5}\right)\;\;\mathrm{with}\;R_{o}=0.01,\;\;Q_{11}=Q_{22}=0.5,Q_{12}=0.49.$$

Here, U(X) denotes a random number drawn from the interval (0,X] with uniform probability, hence also $S_{i}\left(0\right)=O\left(X\right)$. The initialization corresponds to nearly identical student vectors with little prior knowledge. It is inspired by the analyses in [32,34] which showed that the actual value of $R_{o}$ is largely irrelevant for the observed plateau length, while it depends logarithmically on *X* [34]. Corresponding Monte Carlo simulations are shown in Figure 4a for N=500 and randomly drawn initial student vectors, resulting in $R_{im}\left(0\right)=O\left(1/\sqrt{N}\right)$, with $Q_{ik}\left(0\right)$ fixed according to Equation (35). Simulations confirm the theoretical predictions very well, qualitatively.

For very slow drifts of the target concept, the behavior is still similar to the stationary case. For an example with δ=0.005, Figure 4a shows the N→∞ theoretical learning curve and Monte Carlo simulations: After a rapid, initial decrease of the generalization error, a quasi-stationary, unspecialized plateau is reached. Eventually, the symmetry is broken and the system approaches its α→∞ asymptotic state, in which a smaller but non-zero $\epsilon_{g}^{\infty }\left(\delta \right)$ is achieved. Obviously, on-line gradient descent training enables the SCM to track the drifting target to a reasonable degree and maintains a specialized hidden unit configuration. The precise influence of finite size effects on the shape and length of plateaus in Monte Carlo simulations will be studied in greater detail in forthcoming projects.

The behavior changes significantly in the presence of stronger concept drifts: The SCM remains unspecialized even for α→∞ and, consequently, the achievable generalization ability is relatively poor. Figure 4a displays the corresponding learning curve for δ=0.03 as an example, together with the result of a single Monte Carlo simulation.

Figure 4b shows the evolution of the overlap parameters $R_{im}\left(\alpha \right)$ corresponding to the learning curves displayed in Figure 4a. While for δ=0.005 the student units still specialize, the unspecialized plateau state with $R_{im}\approx R$ for all i,m persists for δ=0.03.

**Figure 5:**
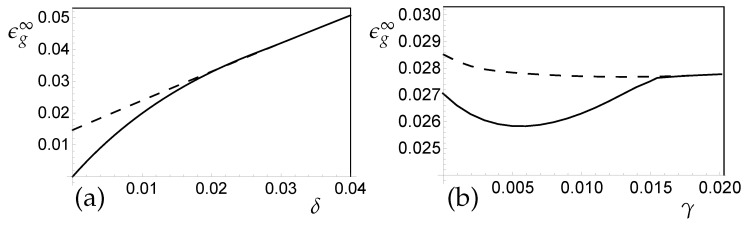
*Regression under Concept Drift: Plateaus and Specialized States*. Soft Committee Machine, regression in the presence of real target drift, learning rate and model parameters as in Figure 4. (**a**) The generalization error vs. the drift parameter δ for γ=0, in the symmetric plateau state with $R_{11}=R_{22}$ and $R_{12}=R_{21}$ (dashed line) and in the α→∞ stationary state (solid). (**b**) The influence of weight decay: For a given drift with δ=0.015, the α→∞ asymptotic generalization error is displayed as a function of the weight decay parameter γ. In addition, the dashed line marks $\epsilon_{g}$ in the unspecialized plateau state.

In Figure 5a, this is illustrated in terms of the (quasi-)stationary values of $\epsilon_{g}$: The system can benefit from the specialization in terms of a low α→∞ asymptotic generalization error (solid line). For δ≈0, the achievable generalization error increases linearly with the drift parameter: $\epsilon_{g}^{\infty }\left(\delta \right)\propto \delta$. Note that $\epsilon_{g}^{\infty }\left(\delta =0\right)=0$ in the perfectly learnable scenario with K=M considered here. On the contrary, for larger δ, the only stable fixed point of the system coincides with an unspecialized configuration (dashed line). The generalization error of the latter also displays a linear dependence on δ for slow drifts.

Weight decay can improve the performance slightly in the presence of weak concept drifts. As displayed in Figure 5a, for an example drift of δ=0.015, the parameter γ in Section 2.4.3 can be tuned to decrease the achievable generalization error in the unspecialized plateau (dashed line) and, more importantly, in the final quasi-stationary tracking state (solid line). Specialization cannot be achieved if the weight decay parameter is set too large. A more detailed analysis of the interplay of learning rate and weight decay will be presented in a forthcoming publication.

## 4. Conclusions

Here, we conclude with a brief summary, provide an outlook on potential follow-up studies and discuss major challenges and open questions.

### 4.1. Brief Summary

In this contribution, we present a modeling framework which facilitates the systematic study and exact mathematical description of on-line learning in the presence of concept drift. The framework is illustrated by the analysis of two model scenarios: The learning of a classification scheme is exemplified in terms of prototype-based Learning Vector Quantization, trained from a stream of clustered input data. Regression problems are addressed in the context of gradient-based training of the Soft Committee Machine, a two-layered feed forward neural network with nonlinear hidden unit activation. Here, the analysis is done in the frame of a student–teacher scenario. In both setups, we study the influence of real drifts, where the target classification or regression scheme are subject to a randomized drift process.

Most importantly, we demonstrate that the presented framework is suitable for the mathematical analysis of a variety of learning and drift scenarios, including weight decay as a possible mechanism of explicit forgetting.

A discussion of the findings in detail is provided in the previous section. In brief, we show that the simple LVQ1 prescription is indeed capable of tracking time-dependent classification schemes in high-dimensional input space under randomized drift. Regression under concept drift displays non-trivial effects in terms of the success of gradient based adaptation in SCM networks. In particular, we observe the drift-induced persistence of unspecialized, sub-optimal plateaus in the learning curve. Thus, on-line learning can display quite different behavior in the presence of concept drift, depending on the underlying target and its properties. In both settings considered here, weight decay has the potential to improve the generalization behavior under drift in the quasi-stationary tracking state.

### 4.2. Future Work and Extensions

In the present contribution, we study only a few, simple scenarios in terms of the considered targets, drift processes and student systems. Several interesting topics can be addressed readily by straightforward modifications of the models:

- The systematic investigation of virtual drifts as in, for instance, non-stationary label noise, prior weights $p_{1,2}$ or cluster separation λ is readily possible by consideration of explicitly time-dependent ODE.
- Alternative LVQ prescriptions, as studied in [39,46,47,48] for stationary data, can be systematically compared in terms of their potential to deal with concept drift.
- Similarly, modifications of the basic gradient descent scheme can be considered under concept drift in the SCM student–teacher scenario (seem for instancem [35,36,38]).
- Deterministic concept drifts, similar to the processes studied in the context of perceptron training in [50,51,52,53], can be considered as well. This way, learning from an *adversary* can be modeled, where the modification of the target depends explicitly on the actual student configuration.
- The restriction to LVQ systems with one prototype per class results, effectively, in the parameterization of linear class boundaries only. This limitation can be lifted by considering distances different from the simple Euclidean measure (see, e.g., [29]). Alternatively, systems with several prototypes per class correspond to non-linear (piece-wise linear) decision boundaries which has non-trivial effects on the training dynamics, as demonstrated for stationary environments in [49].
- Similarly, the investigation of SCM student–teacher scenarios with more general settings of *K* and *M* will provide insight into the interplay of concept drift with the larger number of possible plateau states for K,M>2. Over- and under-fitting effects in mismatched situations with K≠M will be in the center of interest.
- The shallow SCM architectures studied here are limited to a single hidden layer of units. The important extension to deeper networks with several hidden layers will be addressed in forthcoming studies.
- It will be interesting to explore the extent to which the theoretically studied phenomena can be observed in practical situations. To this end, we will investigate the behavior of LVQ and SCM in realistic training set-ups with real world data streams.

### 4.3. Perspectives and Challenges

We have demonstrated that the presented modeling framework bears the promise to provide valuable insights into the effects of concept drift in a variety of learning scenarios. Ultimately, a better understanding of relevant phenomena should facilitate the development and optimization of robust, efficient training algorithms for lifelong machine learning. Variational approaches, as discussed in, for instance [5,6,7,8,35,52,53], could play an important role in this context.

One of the most important challenges, in particular for active methods, is the reliable detection of concept drift in a stream of data. Learning systems should be able to infer not only the nature of the drift (e.g., virtual vs. real), but also estimate its strength in order to tune algorithm parameters such as learning rate or weight decay appropriately. It would be interesting to extend the framework towards such methods, which often rely on the variability of surrogates, such as changes of the observed classification error. The proposed analytical approach would enable us to obtain formal insight into the behavior of the surrogate characteristics in concrete models.

Recently suggested strategies for continual learning include so-called Dedicated Memory Models and the appropriate combination of off-line and on-line learning [21,65,66]. Suitable rejection mechanisms for the mitigation of concept drift were recently considered in [67]. Extensions of our modeling approach in these directions would be highly desirable.

## Abbreviations

The following abbreviations are used in this manuscript:

CLT	Central Limit Theorem
LVQ	Learning Vector Quantization
NPC	Nearest Prototype Classification
ODE	Ordinary Differential Equations
r.h.s.	right hand side
SCM	Soft Committee Machine
WTA	Winner Takes All
